# Letter to the Editor regarding “The relationship between serum vitamin D and fracture risk in the elderly: a meta-analysis”

**DOI:** 10.1186/s13018-020-01811-6

**Published:** 2020-08-07

**Authors:** Qiujiang Li, Xingxia Long, Lijun Cai

**Affiliations:** 1 Graduate School of Ningxia Medical University, Yinchuan, Ningxia Hui Autonomous Region China; 2 Department of Spine Surgery, People’s Hospital of Ningxia Hui Autonomous Region, No. 56, Zhengyuan Street, Yinchuan, 750000 Ningxia Hui Autonomous Region China; 3 Graduate School of Huzhou University, Huzhou, Zhejiang Province China

Dear Editor:

We read with great interest the meta-analysis by Wang et al [1]. entitled “The relationship between serum vitamin D and fracture risk in the elderly: a meta-analysis.” We congratulate the authors for publishing their study in this journal. Yet upon the review of this article, there are serious issues that nullify the conclusions. We seek certain clarifications from the authors.

First, the authors extensively searched the published literature through electronic databases (PubMed and Embase) and manually retrieved the references of the included studies, but these databases seemed to be not enough to retrieve all the eligible studies. Alternatively, other databases do exist, such as Cochrane Library, NLM Gateway, and BIOSIS previews as well as unpublished data like grey literature, which may contribute to get a more comprehensive collection of eligible studies.

Secondly, about the adaptation of systematic review guidelines and registrations, there have been systematic review and meta-analysis protocol registration established that help to maintain a level of homogeneity and quality across all meta-analyses and systematic reviews being conducted. The meta-analysis was performed following the guideline of PRISMA, but the pre-defined protocol was not registered in any platform, such as the Cochrane Library and PROSPERO [2, 3]. Not only do the registration provides transparency in the review, but also improves the quality of conduct of the review and its subsequent reporting.

Thirdly, In total, 20 studies met inclusion criteria and were included in the meta-analysis. Given the characteristics of the study of Swanon et al. [4] in Table 1, such as the study type, number of total fractures, number of hip fractures, country and period, were similar to the study of Cauley et al. [5], we doubt this is the case. Upon close examination, a main reason we found was both of the two studies were case-cohort within the Osteoporotic Fractures in Men Study (MrOS), which recruited 5994 community-dwelling men at six clinical centers in the USA (Birmingham, Alabama, Minneapolis, Minnesota, Palo Alto, California, Monongahela Valley near Pittsburgh, Pennsylvania, Portland, Oregon, and San Diego, California) between March 2000 and April 2002 for a study on musculoskeletal aging. If extracting duplicate data for the sample, it would be likely to lead to an incorrect conclusion, misleading clinical practice. Therefore, in case that several articles from the same trial were published, the study that had the most relevant information or the longest follow-up period might be most appropriate.

Fourthly, Wang et al. [1] revealed that BMI (body mass index) may be a key risk factor to cause osteoporosis and osteoporotic fractures in patients (patients aged 65 and above). Therefore, it is better to perform a subgroup analysis of the low and normal and high BMI in the current study to clear the effect of BMI. Finally, we hope that the authors address the points presented and that the overall discussion of the presented points will only serve to benefit the research community at large.

## Data Availability

Not applicable.

## References

[1] Wang N, Chen Y, Ji J (2020). The relationship between serum vitamin D and fracture risk in the elderly: a meta-analysis. J Orthop Surg Res.

[2] David M, Alessandro L, Jennifer T (2009). Preferred reporting items for systematic reviews and meta-analyses: the PRISMA statement. Ann Intern Med.

[3] Stroup Donna F, Berlin Jesse A, Morto Sally C (2000). Meta-analysis of observational studies in epidemiology: a proposal for reporting. Meta-analysis of observational studies in epidemiology (MOOSE) group. JAMA.

[4] Swanson CM, Srikanth P, Lee CG (2015). Associations of 25-hydroxyvitamin D and 1,25-dihydroxyvitamin D with bone mineral density, bone mineral density change, and incident nonvertebral fracture. J Bone Miner Res.

[5] Cauley JA, Parimi N, Ensrud KE (2010). Serum 25-hydroxyvitamin D and the risk of hip and nonspine fractures in older men. J Bone Miner Res.

